# Ganzheitliche Schulentwicklung zur Implementierung von Educational Technologies in Zeiten der digitalen Transformation – eine Case-Study zum Selbstevaluationstool SELFIE

**DOI:** 10.1007/s42010-022-00155-w

**Published:** 2022-08-09

**Authors:** Olivia Wohlfart, Ingo Wagner

**Affiliations:** Karlsruher Institut für Technologie (KIT), Karlsruhe, Deutschland

**Keywords:** Schulentwicklung, Selbstevaluation, Digitale Medien, Digitalisierung, Case-Study, Educational Technology, School development, Self-evaluation, Digital media, Digitization, Digitalization, Case study, Educational technology

## Abstract

Die digitale Transformation prägt das Bildungssystem und Schulentwicklung zunehmend. Dabei spielt die Implementierung von Educational Technologies in der Schule und im Unterricht eine wichtige Rolle. Systemisch handelt es sich um eine Schulentwicklungsaufgabe, die nachhaltige und kontinuierliche Veränderungen auf allen Ebenen von Schule umfasst und (neue) Herausforderungen an schulische Akteursgruppen stellt. Neben Entwicklungen der Organisations‑, Unterrichts- und Personaldimensionen bedarf es hierfür auch Veränderungen hinsichtlich Technologie- sowie Kooperationsmechanismen spezifisch der Bedürfnisse der Einzelschulen. Mittels einer Case-Study wird in der vorliegenden Studie die Ein- und Durchführung des Selbstevaluationstools SELFIE zur Begleitung in der digitalen Transformation einer Schule multimethodisch analysiert. Die Ergebnisse der Selbstevaluation (*n* = 265) sowie Erkenntnisse aus mehrepisodischen Interviews (*n* = 11) mit verschiedenen schulischen Akteur*innen werden präsentiert und unter Berücksichtigung der Schulentwicklungsdimensionen interpretiert. SELFIE wird dabei zunächst als digitales Tool für die Selbstevaluation in Bezug auf die Implementierung von Educational Technologies kritisch reflektiert und dessen Wirkungen auf die Schulentwicklungsdimensionen diskutiert. Abschließend erfolgt ein Ausblick zu den Potenzialen sowie kritischen Herausforderungen der Selbstevaluation im Hinblick auf Schulentwicklung für Forschung und Praxis.

## Abstract (en)

The digital transformation is increasingly shaping the education system and school development. In this context, the implementation of educational technologies in schools and classrooms plays a critical role. In systemic terms, this is a school development task that involves sustainable and continuous changes at all levels of school and poses (new) challenges for the groups of school stakeholders. In addition to the development of organizational, teaching and personnel dimensions, this also requires changes in the technology and cooperation mechanisms specific to the needs of individual schools. By means of a case study, this study analyzes the implementation and execution of the self-evaluation tool SELFIE at a school using a multimethodological approach. The results of the self-evaluation (*n* = 265) as well as findings from multi-episodic interviews (*n* = 11) with various school actors are presented and interpreted in consideration of the school development dimensions. In the discussion, SELFIE is first critically reflected as an eligible digital tool for self-evaluation in relation to the implementation of educational technologies. Second, its effects on the school development dimensions are discussed. Finally, an outlook on the potentials as well as critical challenges of SELFIE with regard to school development for research and practice is given.

## Einleitung

Eine der aktuell größten und bedeutsamsten Herausforderungen des Bildungssystems in Deutschland ist die digitale Transformation (Heinen und Kerres 2017; KMK 2016; Labusch et al. 2020). Schulen sollen leistungsfähig sein, um Schülerinnen und Schüler (SuS) auf eine digital geprägte Welt vorzubereiten (Ilomäki und Lakkal 2018). Diverse bildungspolitische Dokumente auf europäischer und nationaler Ebene fordern hierbei eine pädagogisch sinnvolle Integration von Educational Technologies (ET) zur Förderung von Digitalkompetenz (BMBF 2016, 2021; Europäische Union (EU) 2021; KMK 2016, 2021). Systemisch handelt es sich bei dem Aufbau und der Entwicklung dieser digitalen Leistungsfähigkeit um eine Schulentwicklungsaufgabe, die in einem ganzheitlichen Verständnis nachhaltige, kontinuierliche Maßnahmen und Veränderungen auf allen Ebenen von Schule sowie (neue) Herausforderungen an die darin befindlichen Akteursgruppen umfasst (Eickelmann und Gerick 2017; Fischer 2015; Heinen und Kerres 2017). Im Rahmen dieser angestrebten digitalen Transformation nimmt die autonome Einzelschule eine herausragende Bedeutung ein (Dalin et al. 1990; Kolbe 2010). Da eine einheitliche Lösung für alle Schulen oder ein starres Vorgehen nicht möglich sind (Rolff 2016), hat es sich als förderlich herausgestellt, Schulen bei diesem sehr individuellen Prozess spezifisch zu unterstützen. Entsprechend fordert auch die KMK (2021, S. 16), „digitalisierungsbezogene Schulentwicklungsprozesse“ auf „Einzelschulebene“ in den Blick zu nehmen.

Die digitale Leistungsfähigkeit einer Schule ist durch kulturelle, politische und infrastrukturelle Rahmenbedingungen geprägt und äußert sich insbesondere durch eine pädagogisch sinnvolle Integration von ET (Costa et al. 2021). Um ET übergreifend und nachhaltig an Schulen zu implementieren ist ein Empowerment-Ansatz besonders erfolgsversprechend (Copland 2003; Schildkamp et al. 2016), der durch Selbstevaluation der digitalen Transformation durch die autonomen Einzelschulen entscheidend unterstützt wird. Ob und wie eine solche Selbstevaluation den Entwicklungsprozess von Schulen in digitaler Transformation tatsächlich positiv beeinflusst, wurde bisher nicht empirisch erforscht. Daher ist es Ziel der vorliegenden Studie, eine Schule in ihrem Selbstevaluationsprozess im Zuge der digitalen Transformation mittels einer Case-Study zu begleiten und dessen Beitrag zur Schulentwicklung zu untersuchen.

## Theoretischer Hintergrund und Forschungsdesiderat

Für eine erfolgreiche digitale Transformation von Schule und Unterricht bedarf es einer pädagogisch und (fach-)didaktisch sinnvollen Integration sogenannter ET (Costa et al. 2021; KMK 2021; Labusch et al. 2020). ET umfassen dabei jegliche Informations- und Kommunikationstechnologien, welche Lehr- und Lernprozesse explizit und/oder implizit unterstützen (Spector 2013). Das Hauptziel der Integration von ET ist dabei eine Verbesserung der Bildungserfahrungen sowie der Lernergebnisse von SuS (Kohler et al. 2022; McKnight et al. 2016). Neben der Implementierung konkreter ET im Unterricht, können diese auch im Rahmen organisatorischer und administrativer Aufgaben im Gesamtkontext Schule Anwendung finden. Die zunehmende Integration von ET in Schulen, gepaart mit hohem gesellschaftlichen, politischen sowie wirtschaftlichen Entwicklungsdruck hin zu Digitalisierung, erfordern eine ganzheitliche, evidenzbasierte Betrachtung und Unterstützung der digitalen Transformation des Bildungssystems (Bromme et al. 2014). Dabei existiert kein regelgeleitetes, festes Vorgehen für die Entwicklung einer Einzelschule, vielmehr ist diese oftmals durch ein „Gemisch von Ideen, Plausibilitäten und Praxisbeispielen“ (Rolff 2016, S. 36) gekennzeichnet. Die Schulentwicklungsforschung steckt im Jahr 2022 noch in Kinderschuhen, denn erst seit den 1990er-Jahren rückte der Fokus vom Schulsystem auf die Einzelschule als „Motor der Entwicklung“ (Dalin et al. 1990). Was früher noch reine Organisationsentwicklungsforschung unter dem Einfluss des New Public Management war, bewegte sich zunehmend zu einer ganzheitlicheren Schulentwicklung mit Fokus auf die Unterrichtsgestaltung unter Berücksichtigung der Lehrkräfte (LK), Klassenführung, Interaktions- und Beziehungsgestaltung (Bollier und Rüttimann 2017). Im deutschsprachigen Raum etablierte sich das praxisstützende Trias-Modell der Schulentwicklungsforschung nach Rolff (Fischer 2016; Holtappels und Feldhoff 2010; Holtappels 2011; Retzl 2014). Die Entwicklung der Einzelschule wird demnach im System zwischen Organisationsentwicklung, Unterrichtsentwicklung und Personalentwicklung eingeordnet (Rolff et al. 2000; Rolff 2010, 2016). Die Ausgestaltung der digitalisierungsbezogenen Schulentwicklung ergänzt diese Entwicklungsdimensionen nun um zwei weitere: die Technologie- und Kooperationsentwicklung (Eickelmann und Gerick 2017). Für eine erfolgreiche und nachhaltige Schulentwicklung bedeutet dies, dass Veränderungen stets im Gesamtkontext der fünf Entwicklungsdimensionen untersucht und eingeordnet werden müssen, da sie zueinander in Wechselwirkung stehen.

1. **Organisationsentwicklung:** Die Organisationsentwicklung zentriert Veränderungen bezüglich Planung, Durchsetzung und Kontrolle (der Schule) in Reaktion auf gesellschaftliche Veränderungen, empirische Bildungsforschung und damit einhergehende bildungspolitische Entscheidungen, bspw. die Integration von ET in die Schule (Eickelmann und Gerick 2017). Organisationskultur und -struktur, Strategie sowie Systemgesetze beeinflussen dabei gleichermaßen das Potenzial und die Geschwindigkeit möglicher Entwicklungsprozesse auf Organisationsebene (Kantelberg und Speidel 2017). Während diese Dimension gestalterisch bei der (erweiterten) Schulleitung (SL) und Schulträgern liegt, sind alle Akteursgruppen von Veränderungsprozessen betroffen (vgl. Prozess von Halbtags- zu Ganztagsschulen bspw. bei Fischer 2015).
2. **Unterrichtsentwicklung:** Auch die Art des Unterrichtens befindet sich in einem konstanten Wandel, beeinflusst durch empirische Bildungsforschung, Bildungsstandards, Lehramtsaus-, -fort- und -weiterbildung sowie gesamtgesellschaftliche Veränderungen. Insbesondere die Entwicklung vom lehrerzentrierten Unterricht hin zu Projektarbeit, Freiarbeit und schülerzentrierten Methoden prägten diese Entwicklungsdimension in den letzten Jahrzehnten (vgl. hierzu Gugel 2007; Meyer 1987; Rolff et al. 2000). Die digitale Transformation beeinflusst Unterricht dabei mit ihrer Dynamik und Schnelllebigkeit im eher trägen System Schule auf besondere Art und Weise. LK können dabei eine treibende Kraft dieser Transformation sein. Die COVID-19-Pandemie und der damit verbundene Fernunterricht zeigten jedoch auch, dass LK teilweise Gefangene der Digitalisierung sind und sich gezwungen fühlen, ET einzusetzen (Wohlfart et al. 2021). Dabei sollten ET im Zuge der digitalen Transformation entsprechend nicht als „Add-On“ verstanden werden, sondern als „integraler Bestandteil im Fachunterricht“ Akzeptanz finden (Eickelmann und Gerick 2017, S. 71). Bisherige Forschung zur Rolle der LK im Prozess der digitalen Transformation konzentriert sich häufig entweder auf ihre (wahrgenommene) digitale Kompetenz oder auf ihre Bereitschaft und Fähigkeit, ET im Unterricht zu integrieren (Wohlfart und Wagner in Vorbereitung). Damit Unterrichtsentwicklung nicht vom Engagement einzelner LK abhängig bleibt, bedarf es einer nachhaltigen und zielgerichteten Integration dieser in die Gesamtschulentwicklung sowie einer Unterstützung der LK durch die SL und die systemischen Rahmenbedingungen (Lorenz et al. 2017). Dieser Weg bedeutet auch, dass angehende LK adäquat vorbereitet werden um ET pädagogisch und (fach-)didaktisch sinnvoll im künftigen Unterricht zu implementieren.
3. **Personalentwicklung: **Personalentwicklung sollte zugleich die Ziele und die Entwicklungserfordernisse der Schulen sowie die individuellen Bedürfnisse und Interessen der LK berücksichtigen. Nach Rolff (2016) werden hier vier einzelne Bestandteile verknüpft: die Personalfortbildung, die Personalführung, die Personalförderung sowie die Persönlichkeitsentwicklung als besonderer Teil des pädagogischen Entwicklungsprozesses. Auch hier können gesellschaftliche Entwicklungen Druck auf personale Entwicklung ausüben (beispielsweise der Shift hin zu einem inklusiven Umgang mit Heterogenität (z. B. Wagner et al. 2021)). Während diese Dimension schulischer Entwicklung hauptsächlich in der Verantwortung der jeweiligen SL liegt, bedarf es auch hierfür auf struktureller Ebene geeigneter Rahmenbedingungen (Fortbildungsangebote für LK; Führungskräftetraining; technische Infrastruktur; Zeit für Fortbildungen). In Bezug auf die digitale Transformation sind in der Personalentwicklung insbesondere geeignete Fortbildungsangebote zur Entwicklung von Digitalkompetenz der LK gefordert (KMK 2021). Auch den potenziellen Vorteilen neuer ET als geeignete Tools im Rahmen der Personalentwicklung wird bisher in deutschen Schulen wenig Beachtung geschenkt (Gerick et al. 2016; Tulowitzki und Gerick 2018).
4. **Technologieentwicklung:** Diese vierte Entwicklungsdimension hat sich in den letzten Jahren parallel zur Organisationsentwicklung etabliert. Sie ist inhaltlich durch Döbeli Honegger (2016) sowie Eickelmann und Gerick (2017) geprägt und umfasst insbesondere IT-Ausstattungskonzepte für Einzelschulen. Der SL wird auch hier ein besonderer Stellenwert in der Ausgestaltung und Umsetzung zugeschrieben. Parallel beeinflussen zunehmend auch bildungspolitische Beschlüsse (bspw. KMK 2016; 2021) und Maßnahmen (bspw. Verwaltungsvereinbarung DigitalPakt Schule; Wohlfart und Wagner (im Druck)) das Potenzial dieser Entwicklungsdimension. Zur pädagogischen und (fach-)didaktischen Integration von ET an Schulen bedarf es neben eines schulspezifischen Medienkonzeptes auch entsprechender schulischer IT-Infrastrukturen (Netzanbindung, Hardware, Software) sowie technischen und pädagogischen IT-Supports (Labusch et al. 2020). Insgesamt erscheinen partizipativ im Kollegium erarbeitete Konzepte zur Technologieentwicklung erfolgsversprechend (Eickelmann und Gerick 2017).
5. **Kooperationsentwicklung: **Die Kooperationsentwicklung zwischen Akteursgruppen der Schulen (LK, SL, SuS, Eltern, Schulträger, Kommunalpolitik etc.) hat sich in den letzten Jahren der Schulentwicklungsforschung ebenfalls als neue Entwicklungsdimension für eine erfolgreiche und nachhaltige Schulentwicklung, insbesondere zwischen LK (fachübergreifend), etabliert (Eickelmann 2010; Drossel et al. 2016; Spiteri und Chang Rundgren 2020). Bezüglich ET wirken Kooperationen zwischen spezifischen Akteuren (bspw. zwischen LK und SuS; Harper 2018) sowie Vernetzungen zwischen Schulen (Eickelmann und Gerick 2017) positiv auf den gesamten Schulentwicklungsprozess. Langfristige und erfolgreiche Kooperationsentwicklungen bedürfen der expliziten Unterstützung der SL.

Veränderungsprozesse wie die digitale Transformation sollten in allen fünf Entwicklungsdimensionen langfristig geplant, sowie wiederholt und kontinuierlich evaluiert werden (Eickelmann und Gerick 2017; Lorenz et al. 2017). Unter Berücksichtigung der spezifischen Kultur der Einzelschulen, Fähigkeiten der darin handelnden Akteursgruppen und vorhandener Infrastruktur entstehen somit individuelle Schulentwicklungsprozesse.

Um die Qualität einer weitestgehend autonom agierenden Schule zu sichern, finden sich verschiedene Konzepte, welche besonders durch externe und interne Evaluationsprozesse gekennzeichnet sind (Kolbe 2010; Rolff und Bastian 2002). Externe Schulevaluation (wie bei der Schulinspektion) analysieren das Management und die Kultur einer Schule, Maßnahmen der Qualitätsentwicklung sowie Erträge einer Schule (Buhren 2019). Die Motivation einer Schule, sich selbst zu evaluieren, ist andererseits besonders durch das Interesse gekennzeichnet, den eigenen Erkenntnis- und Tätigkeitshorizont der Qualitätsentwicklung zu verbessern und zu erweitern (Wendt 2008). Deshalb scheint Selbstevaluation bzw. interne Evaluation an Schulen hinsichtlich Veränderungen wirksamer und nachhaltiger zu sein als externe Evaluation (Burkard und Müller 2019; Riecke-Baulecke 2008). Auch wird Schulen im Prozess der digitalen Transformation ein digitales Self-Assessment nahegelegt (KMK 2021). Diese sind besonders dann effektiv, wenn sie (1) auf der Grundlage validierter und theoriegestützter Instrumente angewandt werden, (2) alle schulischen Akteursperspektiven berücksichtigen und (3) evidenzbasierte Maßnahmen für Schulentwicklung auf mehreren Ebenen ermöglichen (Antoniou et al. 2016).

Während bisherige Studien primär den allgemeinen Einsatz von Evaluationen an Schulen anstelle der Wirksamkeit oder des Einflusses auf die Schulentwicklung fokussierten (Burkard und Müller 2019), ist es naheliegend, dass insbesondere interne Evaluationsprozesse geeignet sind, um Schulen in Veränderungsprozessen zu begleiten und dabei ihre Selbstregulation zu unterstützen. Ziel dieser Studie ist es daher, die Wirkungen eines Evaluationsprozesses nach Buhren (2019) durch die Einführung eines Selbstevaluationsinstruments auf die digitale Transformation einer Schule zu analysieren. Im Vordergrund der Studie stehen folgende Forschungsfragen (FF):

### FF1

Welche Wahrnehmungen, Einschätzungen und Nutzungen zeigen sich durch ein Selbstevaluationsinstrument hinsichtlich des Status-Quo zum Einsatz von ET in einer Schule?

### FF2

Welche Erwartungen haben schulische Akteursgruppen hinsichtlich der Implementierung von Selbstevaluationstools und welche Veränderungsprozesse können aus ihrer Sicht durch diese angestoßen werden?

### FF3

Wie wirkt sich die Ein- und Durchführung einer Selbstevaluation hinsichtlich der Implementierung von ET auf die fünf Entwicklungsdimensionen der Schulentwicklung aus?

## Methodik

**Figure 1:**
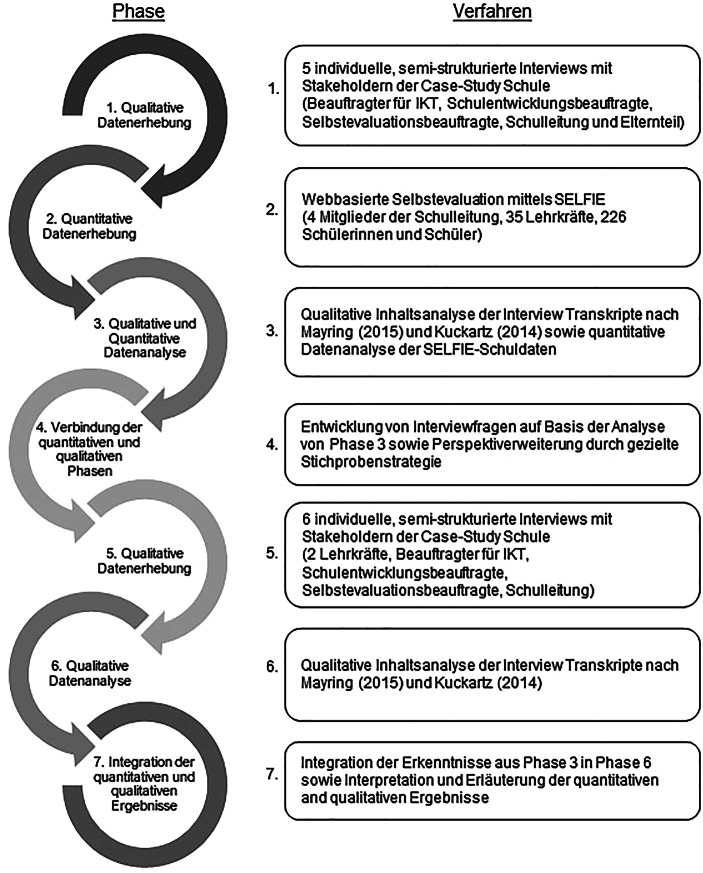

Mittels einer umfangreichen Case-Study wurde die Ein- und Durchführung einer Selbstevaluation an einer Schule multimethodisch begleitet und analysiert. Die explorative Untersuchung der Einzelschule im Rahmen einer Case-Study erscheint vor dem Hintergrund des aufgezeigten Forschungsdesiderats besonders zielführend, um Forschungserkenntnisse für die Praxis sowie künftige Forschung ableiten zu können. Das Case-Study-Design ermöglicht dabei die Untersuchung eines realen und aktuellen Falls und berücksichtigt zusätzlich die Vielzahl kontextueller Bedingungen (Yin 2018). Schlüsselelemente einer Case-Study sind neben der Fokussierung auf eine abgegrenzte Einheit, die Anwendung von mindestens zwei Datenerhebungsmethoden und/oder die Berücksichtigung von mindestens zwei verschiedenen Perspektiven (Hamilton und Corbett-Whittier 2013). Um der Komplexität gerecht zu werden, teilt sich die vorliegende Studie in sieben sequenzielle Phasen und berücksichtigt dabei die Perspektiven verschiedener Akteursgruppen (siehe Tab. 2 in Abschn. 3.3). Abb. 1 zeigt die einzelnen Phasen sowie angewandten Verfahren der Studie auf. Die Durchführung der Selbstevaluation mittels SELFIE wird hierbei von semi-strukturierten Interviews mit unterschiedlichen Interviewpartner*innen flankiert. Im Folgenden wird zunächst die Case-Study-Schule vorgestellt, gefolgt von der Informationen zur Wahl und Erläuterungen der gewählten Forschungsinstrumente.

### Die Case-Study-Schule

Mittels eines persönlichen Kontaktes konnte eine Case-Study-Schule, welche sich neben Forschungserkenntnissen für die Wissenschaft auch wichtige Folgerungen für ihre individuelle Schulentwicklung erhoffte, für die umfangreiche und aufwendige Untersuchung gewonnen werden. Die Case-Study-Schule ist eine berufliche Schule in einer mittelgroßen Stadt im Nordwesten Baden-Württembergs. Hier wird – laut eigenen Angaben der Schule – eine vielfältige und „bunte“ Schülerschaft von 1400 SuS von ca. 120 LK innerhalb von fünf Berufsprofilen unterrichtet. Die erweiterte SL besteht aus acht Personen. Neben der engen Kooperation mit Praxisstellen in der Erzieher- und Altenpflegeausbildung kooperiert die Case-Study-Schule mit ausgewählten Unternehmen und Schulen mit dem Ziel, SuS besondere Praxiserfahrungen zu ermöglichen. Die Schule engagiert sich seit 2003 im Rahmen verschiedener Schulentwicklungskonzepte in den Bereichen Selbständigkeit und Qualitätsmanagement, zudem ist sie seit 2012 zunehmend mit Selbstevaluation als Steuerungselement von Schulentwicklung beschäftigt. Digitale Transformation ist ein besonderes Thema an dieser technisch sehr gut ausgestatteten Schule. Aktuell wird beispielsweise an einem integralen Medienkonzept gearbeitet, welches die Heterogenität der SuS sowie damit einhergehende Individualisierung von Lernen und eine Veränderung von Rollenverhältnissen in der Schule berücksichtigt.

### Instrumente

Im Rahmen der vorliegenden Case-Study wurden parallel zur Durchführung einer quantitativen Selbstevaluation mittels vorhandenem Instrument SELFIE, mehrepisodische, qualitative Interviews mit verschiedenen schulischen Stakeholdern (SL, IKT-Beauftragter, LK, Eltern) geführt. Dabei ermöglicht das Case-Study-Design explizit die Berücksichtigung der Erkenntnisse vorheriger Phasen bei der Auswahl der Interviewpartner*innen sowie eine flexible Gestaltung der Datenerhebungsinstrumente (wie Interviewleitfäden) (Creswell et al. 2003).

#### SELFIE

Aus der großen Zahl an vorhandenen Selbstevaluationsinstrumenten (bspw. IFS Schulbarometer, SEIS, QUES, SEP) wurde das digitale SELFIE-Tool (Self-reflection on Effective Learning by Fostering the Use of Innovative ET) gewählt. Das Tool ist aus einer der elf Initiativen der EU-Kommission entstanden, die im Aktionsplan für digitale Bildung (EU 2020) festgehalten wurden und basiert auf dem theoretischen Modell des *European Framework for Digitally Competent Educational Organisations *(DigCompOrg 2022; Kampylis et al. 2015). Das Modell umfasst Aspekte und Prozesse, die für eine erfolgreiche Integration von digitalem Lernen in Bildungsorganisationen relevant sind (Meier 2019).

Das Tool wurde aus verschiedenen Gründen für die vorliegende Studie gewählt. Zum einen bietet das Instrument eine validierte und theoriegestützte Grundlage für eine effektive Selbstevaluation (Antoniou et al. 2016; Costa et al. 2021). Zusätzlich fokussiert SELFIE als Selbstevaluationstool den Einsatz von ET in der Schule und kann somit die digitale Leistungsfähigkeit der Schule einordnen (Costa et al. 2021; Tulowitzki et al. 2021, S. 20; vgl. Forschungsfragen). Dabei integriert und differenziert das Tool zusätzlich die Perspektiven der verschiedenen schulischen Akteursgruppen. Zudem entsprechen die Fragen und Bereiche von SELFIE auch den Zielsetzungen und Anforderungen der Case-Study-Schule hinsichtlich digitaler Transformation, sodass auch auf praktischer Ebene eine Passung und ein Mehrwert erwartbar sind.

SELFIE als Tool basiert auf einer ausführlichen Auseinandersetzung mit dem DigCompOrg Framework, einer Meta-Analyse bestehender Instrumente, welche die Nutzung von Technologien in Schulen fokussieren (Kampylis et al. 2015), einer ausführlichen Testphase des daraus entstandenen SELFIE-Prototyps (Castaño-Muñoz et al. 2018) und letztendlich einer psychometrischen Validierung der Reliabilität und internen Konsistenz des finalen Instruments (Costa et al. 2021). Ziel dieses Selbstevaluationstools ist es, Stärken und Schwächen bei der Integration von ET im Unterricht, von ET beim Lernen und von ET bei der Bewertung zu ermitteln, um eine interne, evidenzbasierte Debatte der Einzelschule hinsichtlich ihrer digitalen Leistungsfähigkeit zu initiieren und zu fördern (Costa et al. 2021; SELFIE 2022). Das Instrument differenziert dabei mittels drei digitaler Fragebögen je nach Akteursgruppe (SL, LK und SuS), welche Fragen in Bezug auf bis zu acht Bereiche beantwortet werden müssen (siehe Tab. 1)^1^.

**Table 1:** 

Bereich	Items für Schulleitungsmitglieder	Items für Lehrkräfte	Items für Schülerinnen und Schüler
Schulleitung	6	6	0
Zusammenarbeit und Vernetzung	6	6	1
Infrastruktur und Ausstattung	15	15	10
Berufliche Weiterbildung	4	4	0
Pädagogik: Unterstützung und Ressourcen	5	5	1
Pädagogik: Umsetzung im Klassenzimmer	6	6	8
Bewertungsverfahren	9	9	9
Digitale Kompetenz der SuS	10	11	18
Insgesamt	61	62	47

Jede der Fragen wird durch eine aufsteigende Likert-Bewertungsskala hinsichtlich Zustimmung zu bestimmten Aussagen von eins bis fünf (1 = stimme überhaupt nicht zu; 5 = stimme voll und ganz zu; sowie „keine Angabe“) beantwortet. Auf Grundlage der Antworten wird nach Beendigung der Umfrage automatisch ein Schulbericht für die teilnehmende Schule erstellt, durch welchen die Stärken und Schwächen in den verschiedenen Bereichen deutlich werden. Eine weitere Eigenschaft von SELFIE ist, dass das Tool in angepassten Versionen von allen Grundschulen, Schulen der Sekundarstufe I und II sowie Berufsschulen in Europa genutzt werden kann. Zusätzlich besteht für die Einzelschulen die Möglichkeit, weitere Fragen zu ergänzen oder auf (Teil‑)Bereiche zu verzichten.

#### Leitfadeninterviews

Um einen ganzheitlichen Blick auf den Selbstevaluationsprozess zu erhalten, wurden mehrepisodische Interviews mit ausgewählten Akteuren der Schule durchgeführt und ausgewertet. Das semi-strukturierte Interviewformat ermöglicht ein detailliertes Verständnis von Themen und sozialen Kontexten und bietet ein gewisses Maß an Flexibilität im Interviewprozess, je nach Hintergrund, Erfahrung und Status der Befragten (Denzin und Lincoln 2011). Unter Berücksichtigung des theoretischen Hintergrunds wurden hierfür zunächst spezifische, semi-strukturierte Interviewleitfäden für die einzelnen Akteursgruppen zu Beginn des Prozesses entwickelt. Hauptfokus der ersten Reihe von Interviews war es, einen Status-Quo der einzelnen Akteursgruppen hinsichtlich Erfahrungen bezüglich der Integration von ET sowie Erwartungen an SELFIE vor der Durchführung zu erfassen. Mittels variierender Schwerpunkte der Leitfäden wurde den verschiedenen Perspektiven der befragten Personen Rechnung getragen. So fokussierte der Leitfaden für die Schulentwicklungsbeauftragte beispielsweise konkret zu erwartende Schulentwicklungsprozesse, während der Leitfaden für die Mutter stärker ihre Ansicht zum Status-Quo der aktuellen digitalen Leistungsfähigkeit der Schule betonte. Nach Betrachtung des automatisierten SELFIE-Berichts sowie der Erkenntnisse der ersten Interviewreihe, wurden erneut akteursspezifische Interviewleitfäden entwickelt. Diese zweite Runde semi-strukturierter Interviews fokussierte neben einer Einschätzung hinsichtlich der Ergebnisse des Berichts, den Prozesscharakter der Selbstevaluation bezüglich des persönlichen Befindens der Teilnehmenden während und im Anschluss an die Teilnahme sowie damit einhergehenden Erwartungen an künftige Entwicklungsschritte.

Die 11 Interviews dauerten zwischen 14 und 28 min, wurden audiovisuell aufgezeichnet und nach einem Transkriptionsleitfaden in Anlehnung an Dresing und Pehl (2018) inhaltlich-semantisch transkribiert. Nonverbale Signale wurden bewusst weggelassen, um die Lesbarkeit der Transkripte zu verbessern. Darüber hinaus wurden die Transkripte einer Anonymisierung unterzogen, um zu vermeiden, dass bestimmte Aussagen auf einzelne Personen zurückgeführt werden können. Insgesamt entstanden aus den Interviews 59 Seiten einzeiligen transkribierten Textes, und die Gesamtzeit der Interviews betrug 223 min.

### Stichprobe

An der Selbstevaluation mit SELFIE haben im Zeitraum vom 7.–18. Juni 2021 insgesamt vier Personen aus der (erweiterten) SL, 35 LK und 226 Schüler*innen teilgenommen. Zusätzlich wurden sieben Personen zu mindestens zwei Zeitpunkten (03.05.–27.07.2021) mittels semi-strukturierter Interviews hinsichtlich der Haltung und Wirkung des Selbstevaluationsprozesses befragt (siehe Tab. 2 für eine Übersicht der Interviewpartner*innen inklusiv pseudonymisierter Bezeichnung).

**Table 2:** 

Pseudonym	Rolle im Rahmen der Case Study	Fächer	Berufserfahrung (in Jahren)	Geschlecht	Interviewzeitpunkt(e)
IKT	Beauftragter für IKT	Informatik & Wirtschaftslehre	11	Männlich	Pre & Post
LK1	Lehrkraft	Deutsch & Biologie	2	Weiblich	Post
LK2	Lehrkraft	Mathematik & Italienisch	6	Weiblich	Post
M	Elternteil eines Schülers	–	–	Weiblich	Pre
SEB	Schulentwicklungsbeauftragte	Philosophie & Ethik	23	Weiblich	Pre & Post
SEVA	Selbstevaluationsbeauftragte	Informatik & Mathematik	11	Weiblich	Pre & Post
SL	Schulleitung	Volks- & Betriebswirtschaftslehre	21	Männlich	Pre & Post

Die Teilnehmer*innen der ersten Interviewreihe wurden mittels gezielter Stichprobenstrategie aufgrund ihrer Rollen im Schulentwicklungsprozess ausgewählt. Im Sinne des Case-Study-Designs wurde dabei besonderer Wert auf eine Vielfalt verschiedener Perspektiven beteiligter Akteursgruppen gelegt. Somit wurden zunächst der Schulleiter, die Leiterin des Schulentwicklungsteams, die Selbstevaluationsbeauftrage und der Beauftragte für Informations- und Kommunikationstechnologien befragt. Diese Personen zählen aus Sicht der begleiteten Schule zu den Relevantesten für den Schulentwicklungsprozess. Ergänzend hierzu wurde in der ersten Interviewreihe eine engagierte Mutter befragt.

In der zweiten Interviewreihe wurden, bis auf jene Mutter (aufgrund von zeitlichen Engpässen ihrerseits), alle zuvor genannten Personen erneut interviewt. Hierbei wurden die Ergebnisse des SELFIE-Berichts sowie Erkenntnisse der ersten Interviews durch die Verbindung der quantitativen und qualitativen Datenerhebungsphasen (siehe Abb. 1) explizit berücksichtigt. Um die Vielfalt an Perspektiven weiter zu ergänzen, wurden in der zweiten Interviewrunde gezielt zwei vom Schulentwicklungsprozess losgelöste Lehrerinnen (mit unterschiedlichen Fächern) befragt.

### Datenauswertung

Die Datenauswertung erfolgte zunächst getrennt für die erste Interviewreihe sowie den Ergebnissen des SELFIE-Berichts. Die transkribierten Interviews wurden mittels qualitativer Inhaltsanalyse nach Mayring (2015) und Kuckartz (2014) unter Berücksichtigung der Schulentwicklungsdimensionen ausgewertet. Hierfür wurden die Transkripte wiederholt von zwei Forscher*innen gelesen und Abschnitte mit MAXQDA Analytics Pro 2020 gemäß deduktiver Kategorien (z. B. Entwicklungsdimensionen, Veränderungsprozesse usw.), sowie induktiver (z. B. 5‑Jahres-Ziele, Erwartungen usw.) Kategorien kodiert. Die zuvor festgelegten deduktiven Kategorien wurden in einer dreiköpfigen Forschungsgruppe entwickelt. Eine erste Kodierungsrunde durch ein*en Forscher*in wurde durch eine zweite Kodierungsrunde durch die Erstautorin ergänzt. Die für die Kodierung zuständigen Forscher*innen standen zudem über den gesamten Kodierungsprozess im engen und kritisch-reflektierenden Austausch.

Aufgrund fehlenden Zugangs zu den Rohdaten der Onlinebefragung, werden die Ergebnisse des automatisierten SELFIE-Berichts deskriptiv dargestellt und prägnante Ergebnisse hervorgehoben. Im Sinne der Verbindung der quantitativen und qualitativen Phasen wurden bei der Konzipierung der zweiten Interviewreihe die Ergebnisse des SELFIE-Berichts und Erkenntnisse der ersten Interviewreihe explizit berücksichtigt. Die Transkripte der zweiten Interviewreihe wurden analog zur ersten Reihe von zwei Forscher*innen mittels qualitativer Inhaltsanalyse ausgewertet. Insgesamt wurden somit für alle elf Interviews 635 Segmente in 5 Hauptkategorien und 32 Unterkategorien mit einer Spannweite von 33 (Mutter) bis 136 (SL) kodiert. Tab. 3 im Anhang gibt einen Überblick über die Hauptkategorien, Unterkategorien sowie zugehörigen Definitionen und Ankerbeispiele. Um die Güte des gesamten Forschungs- und Datenanalyseprozesses zu gewährleisten, wurde zusätzlich die von Smith und McGannon (2018) empfohlene Methode der „critical friends“ in Verbindung mit Selbstreflexivität eingesetzt. Zu diesem Zwecke fanden zwischen den einzelnen Datenerhebungs- und -analysephasen mehrere inhaltliche Treffen innerhalb der Forschungsgruppe statt. Dies bot Gelegenheiten für einen reichhaltigen Dialog über Interpretationsmöglichkeiten und verlangte von den Forschenden, dass sie ihre Gedankengänge explizit machten (Smith und Hodkinson 2009). Die Rolle eines „critical friend“ besteht dabei nicht darin, ‚zuzustimmen‘ oder einen Konsens zu erreichen, sondern vielmehr darin, Reflexivität zu fördern, indem die Wissenskonstruktion des anderen in Frage gestellt wird. Für die Ergebnispräsentation und Interpretation hinsichtlich der Forschungsfragen, wurden Zitate aus den Interviewtranskripten extrahiert. Bei denjenigen Interviewpartner*innen, die zweifach befragt wurden, werden die Zitate nach dem Pseudonym mit „a“ für die erste Interviewreihe sowie „b“ für die zweite Interviewreihe gekennzeichnet. Zusätzlich werden die aus der Datenanalyse herausgearbeiteten Kategorien zur Transparenz bei der Darstellung kursiv geschrieben.

## Ergebnisse

Zunächst wird der Status-Quo bezüglich des Einsatzes von ET der Einzelschule mittels des automatisierten SELFIE-Berichts zusammengefasst und präsentiert (Abschn. 4.1; FF1). Darauf folgt ein Abschnitt zu den verschiedenen Perspektiven schulischer Akteursgruppen sowie angestoßenen Veränderungsprozessen bezüglich der Implementierung eines Selbstevaluationstools mit Schwerpunkt auf die digitale Leistungsfähigkeit der eigenen Schule (Abschn. 4.2; FF2). Abschließend wird der Zusammenhang der Ergebnisse des SELFIE-Berichts und der Interviews mit den fünf Dimensionen der Schulentwicklung präsentiert (Abschn. 4.3; FF3).

### SELFIE und der Status-Quo zum Einsatz von ET

**Figure 2:**
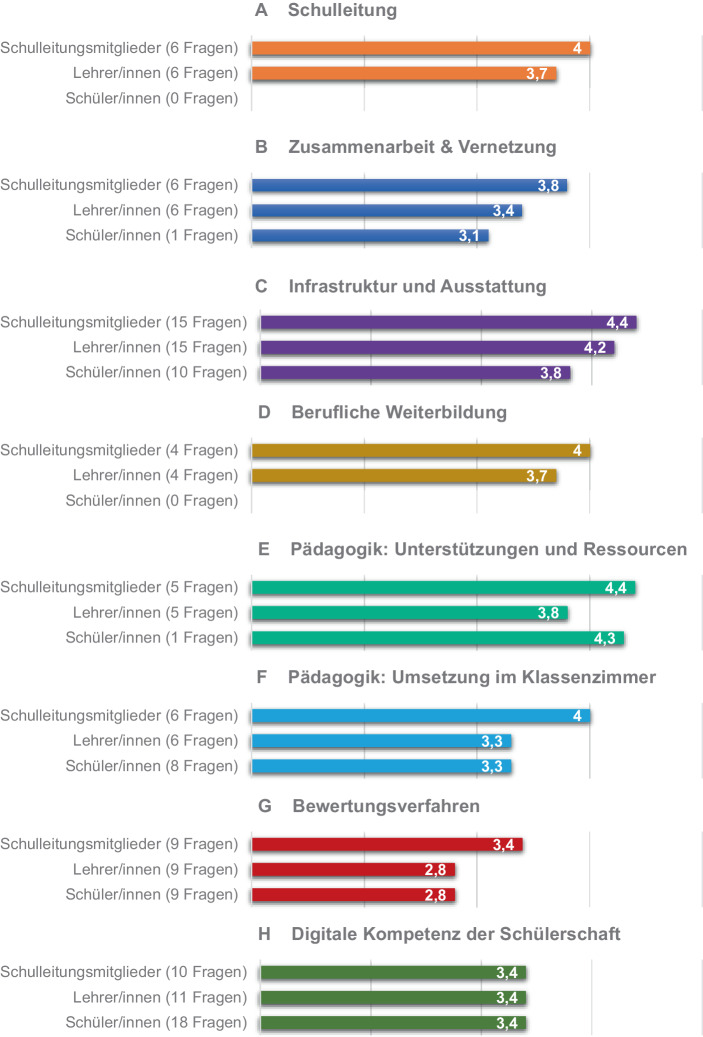

Im Rahmen der Selbstevaluation wurden Fragen zu acht schulischen Bereichen gestellt, welche automatisiert ausgewertet und in Form eines Staus-Quo-Berichts an die Schule übermittelt wurden (vgl. Tab. 1 in Abschn. 3.2.1). Abb. 2 gibt einen Überblick über die Ergebnisse für die befragten Akteursgruppen (SL (*n* = 4), LK (*n* = 35) und SuS (*n* = 226)) auf einer Skala von 1–5 (1 = stimme überhaupt nicht zu; 5 = stimme voll und ganz zu).

Insgesamt fällt die Selbstevaluation, gemessen am theoretischen Mittelwert von 3, überwiegend positiv aus. Der SELFIE-Bericht zeigt zudem einige Subgruppenunterschiede zwischen den Akteursgruppen auf.

Die Mitglieder der SL bewerten sieben der acht Bereiche mit Abstand positiver als die LK und SuS. Insgesamt beantworten die SuS die Fragen etwas kritischer, wobei der Bereich „Digitale Kompetenz der Schülerschaft“ von allen Akteursgruppen gleich bewertet wurde. Im Detail zeigen sich beispielsweise bei dem Item „technische Unterstützung“ (Bereich 3: Infrastruktur und Ausstattung) größere Unterschiede zwischen den Akteursgruppen: Die Schulleitungsmitglieder bewerten diese im Durchschnitt mit 4,8, die LK mit 4,5 und die SuS mit 3,9. Unterschiede existieren in diesem Bereich zusätzlich bei zwei weiteren Items: „Digitale Kluft: Maßnahmen zur Identifizierung von Herausforderungen“ (SL: MW = 4,5; LK: MW = 3,5) und „Digitale Kluft: Unterstützung bei der Bewältigung von Herausforderungen“ (SL: MW = 4,5; LK MW = 3,4). Das Item „fächerübergreifende Projekte“ im Bereich Pädagogik wurde von allen Akteursgruppen als relativ gering ausgeprägt bewertet (SL: MW = 3,5; LK: MW = 2,4; SuS: MW = 3,0). Aufgrund fehlender Rohdaten konnten diese Unterschiede jedoch nicht auf Signifikanz oder Effektstärke getestet werden und sollten demnach nur unter Vorbehalt interpretiert werden.

Die Implementierung von ET nimmt einen besonderen Stellenwert in der Selbstevaluation ein. Demnach geben alle Schulleitungsmitglieder und 71,4 % der LK an, dass Zeitmangel der hauptsächliche Hinderungsgrund bei der Implementierung von ET sei, gefolgt von fehlenden finanziellen Mitteln (50 % der SL), Beschränkung des Schulraums (50 % der SL) sowie geringer digitaler Kompetenz der Lehrkräfte (34,3 % der LK). Hinsichtlich des Umgangs mit Technologien geben 91 % der LK an, sich sicher oder sehr sicher beim Einsatz dieser zu Kommunikationszwecken zu fühlen (74 % im Klassenunterricht, 73 % zur Unterrichtsvorbereitung und 64 % als Mittel für Feedback und Unterstützung). Ein großer Teil (67 %) des Kollegiums gibt an, 76–100 % der Unterrichtszeit mit ET zu gestalten. Im Gegensatz hierzu äußerten 47 % der SuS, mindestens 1 h Technologien in der Schule zu nutzen.

**Figure 3:**
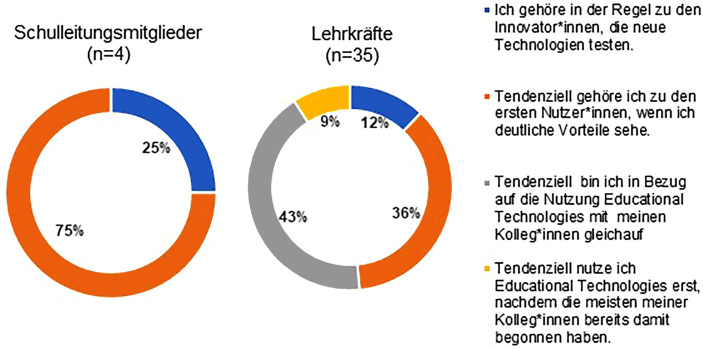

Insgesamt spiegeln die Ergebnisse (überwiegend) positive Einstellungen zur Nutzung von Technologie der SL und LK sowie ein damit einhergehendes Selbstvertrauen bei der Anwendung von ET wider (siehe Abb. 3).

### Erwartungen und Veränderungsprozesse

Die verschiedenen Akteursgruppen wurden mittels semi-strukturierter Interviews hinsichtlich *Erwartungen* (Phase 1) sowie zu durch SELFIE angestoßenen *Veränderungsprozessen* in Bezug auf die *Implementierung von ET* (Phase 5) befragt (FF2). Es zeigte sich, dass die Durchführung von SELFIE von den Befragten als eine Art Vertretungstool der eigenen Selbstevaluation in den Schulentwicklungsprozess eingeordnet und von den verschiedenen Akteursgruppen mit großer Offenheit akzeptiert wurde. Die Schule orientiert sich nach Aussagen der SL und des SEB an dem iterativen „PDSA Zyklus“ (Plan-Do-Study-Act), in welchem SELFIE den Baustein „study“ vertrete, ganzheitlich berücksichtigt wird und somit „einen ganz festen Platz in einem kontinuierlichen Verbesserungsprozess von Schule [hat]“ (SEBa, 207ff.). Die *Erwartungshaltung* der Befragten war vor der Durchführung ergebnisoffen und neugierig, was dieses neue Tool für die digitale Transformation der eigenen Schule bedeuten könne (IKT, SEVA, SL). Im Gespräch verwiesen die meisten Interviewpartner*innen auf den schulspezifischen *5‑Jahres-Plan*. Die Schulleitung fasste die *Erwartungen* an SELFIE wie folgt zusammen:

SELFIE [ist] ein Medium […] und kein Selbstzweck. Das heißt auch hier werden wir schauen müssen, worin bestehen unsere Ziele und wobei kann uns SELFIE […] helfen? (SLa, 237)

Die Implementierung eines digitalen Selbstevaluationstools wie SELFIE wurde als positiver Schritt innerhalb der *Organisationsentwicklung* (M) und als Stärke der eigenen Schule bei der *Implementierung von ET* hervorgehoben (SL, IKT). Viele fassten die Ergebnisse für sich im Abgleich mit ihren Erfahrungen mit ET als Bestätigung so zusammen, dass sie selbst sowie die Schule auf einem richtigen Weg seien, um *Schulentwicklung* hinsichtlich der digitalen Transformation weiter zu vollziehen wie ein Zitat des IKT-Beauftragten zeigt:

Also das jetzt die Infrastruktur gut ausgebaut ist und die technische Ausstattung der Schule gut ist bzw. hoch gerated [bewertet] wurde hat mich jetzt tatsächlich weniger verwundert. (IKTb, 84 ff.)

Nach der ersten Durchführung von SELFIE waren die Befragten etwas reservierter gegenüber dem *Veränderungspotenzial* aus den Erkenntnissen (SEB, SEVA, SL). Positiv wurde dem *Ergebnisbericht* eine unterstützende Wirkung im Sinne einer Datengrundlage zugeschrieben, „um Entwicklung zu forcieren oder zu verwerfen“ (SEBb, 151f.). Dennoch bestand größtenteils Einigkeit unter den Befragten, dass der Mehraufwand des Digitalisierungstools im Vergleich zum tatsächlichen Nutzen im Gesamtkontext von *Schulentwicklung* nicht im Verhältnis stehe und die Option, dafür die schuleigene Evaluation „in die Tonne [zu] klopfen“ (SEBb, 201), eher unwahrscheinlich wäre. Eine langfristige Übernahme des Tools in den Qualitätsmanagementprozess war den Aussagen folgend nicht geplant, da es hinsichtlich *Möglichkeiten* „eben nicht so flexibel anpassbar ist wie [das] selbstgestrickte Selbstevaluationsinstrument“ (SEBb, 217f.). Mit Blick auf zukünftige *Veränderungsprozesse* wurde demnach vorgeschlagen, spezifische Bereiche oder Fragen von SELFIE in die bestehende schuleigene Evaluation zu übernehmen (SL, SEB, SEVA). Befragte verwiesen rückblickend vermehrt und wiederholt auf vergangene *digitale Entwicklungen* mit bereits angestoßenen Projekten und Ideen sowie den Digitalisierungsanschub durch die Covid-19-Pandemie, ohne welchen die Selbstevaluation „wohl etwas irrelevanter gewesen“ wäre (LK2). Die mehrperspektivische Betrachtung durch verschiedene Akteursgruppen wurde in den Interviews gegenüber bisherigen Selbstevaluationstools als *Stärke* hervorgehoben (LK1, IKT, SL). Hinsichtlich *zukünftigen Nutzens* müsse sich die dafür zuständige Schulentwicklungsgruppe mit den aktuellen Zielen und dem Bezug von SELFIE zu diesen auseinandersetzen und ausloten, welche Maßnahmen umgesetzt werden können und müssen (SL). Hierbei stehe die Diskussion des *SELFIE-Berichts* im Vordergrund, dessen Erkenntnisse als Grundlage in der Entwicklung und Überarbeitung des schuleigenen Mediencurriculums berücksichtigt werden, um „den LK oder dem Kollegium [zu] begründen, warum man bestimmte Ziele verfolgt“ (IKTb, 182f.).

Auf persönlicher Ebene reflektierten die Befragten in der zweiten Interviewstudie insbesondere den eigenen Unterricht und damit einhergehendes *Entwicklungspotenzial* und Spielräume hinsichtlich der Implementierung von ET. Hierbei wurden die Bereiche der Bewertungsverfahren sowie der Feedbackkultur als potenzielle Schwächen hinterfragt und individuelle Vorsätze hinsichtlich der Weiterentwicklung in diesen Bereichen formuliert, wie die Aussage einer Lehrkraft illustriert:

Ich habe aber auch Ideen bekommen […], was man da alles machen kann. […], dass man zum Beispiel messen kann, wie oft man mündlich was gesagt hat. […] Das habe ich dann dadurch so ein bisschen erfahren und hab’s dann aber auch mal durchgeführt und habe eben neue Ideen bekommen. (LK1, 18 ff.)

Auch zeigten sich teilweise Widerstände und Frustration in Reaktion auf die Ergebnisse. Zum einen sprachen im Sinne möglicher Schwachstellen bei der weiteren Implementierung von ET mehrere Befragte die *Diskrepanz* zwischen Antwortverhalten der SL im Vergleich zu den LK und SuS an (IKT, LK1, LK2; s. a. Abschn. 4.1).

Also ich finde es schade, weil da muss dann irgendwo eine Diskrepanz sein bei der Wahrnehmung […] und das finde ich witzig, dass das dann auf Schulleitungsebene alles als super positiv empfunden wird. […] ich kann vieles ganz gut nach außen verkaufen, aber ob’s dann tatsächlich in der Praxis so auch funktioniert, das glaube ich ist genau dieser Punkt, der nicht passt. (LK2, 105ff.)

Zum anderen erzählte ein Befragter hinsichtlich *emotionalen Befindens* über Frustration in der Reflexion eigener Erfahrungen durch SELFIE hinsichtlich der nie enden wollenden Vielfalt an Möglichkeiten und Erwartungen bei der Implementierung von ET (LK1), was als mögliche Herausforderung wahrgenommen wurde.

### Dimensionen der Schulentwicklung

Mittels qualitativer Inhaltsanalyse der Interviews wurden Auswirkungen durch die Durchführung von SELFIE für alle fünf Dimensionen der Schulentwicklung (FF3) identifiziert.

#### Organisationsentwicklung

In den Interviews wurden verschiedene Sichtweisen und Geschehnisse der letzten Jahre in Bezug auf die Organisationsentwicklung der Schule präsentiert. Einigkeit herrscht über die Unausweichlichkeit von Veränderung durch die digitale Transformation. Die SL betonte dabei:

wenn klassische didaktische Bildungselemente und Kompetenzen aufgebaut werden müssen, müssen wir auch schulorganisatorisch mittelfristig bereit sein und umdenken. (SLa, 190ff.)

Hierzu soll künftig die Nutzung des Qualitätsmanagement-Systems (SL), digitale Projektplanungstools sowie die nachhaltige Verankerung des Mediencurriculums für das Lehren und Lernen mit ET an der Schule stärker fokussiert werden (IKT). Die SL hob dabei, hervor, dass auf organisatorischer Ebene, „begrenzte Mittel aber […] ein hohes Maß an Entscheidungs- und auch Verwendungshoheit“ klar vor „unbegrenzten Mitteln“ stehe (SLa, 302ff.). Auch die Verpflichtung zur Implementierung spezifischer ET im Unterricht wurde durch die Ergebnisse von SELFIE durch eine LK legitimiert:

Eine andere Möglichkeit wäre zum Beispiel, sich für zwei, drei [ET] zu entscheiden, die es gibt, die man einführt, schulweit, und dann doch mal sagen, jeder muss [diese] zweimal im Jahr anwenden. (LK2, 139ff.)

Die befragten LK und das Elternteil äußerten sich positiv über die Durchführung regelmäßiger Selbstevaluationen im Rahmen des Qualitätsmanagements und der Schulentwicklung und zeigten eine große Offenheit gegenüber SELFIE als neues Instrument mit Fokus auf Digitalisierung. Dies wurde in Zusammenhang mit der Covid-19-Pandemie und zunehmendem Bewusstsein gegenüber dem Nutzen von ET gebracht. Demnach wird die Schule insgesamt verändert aus der Pandemie herauskommen, mit dem Ziel, die Dinge, die „gut liefen“, in der „neuen Schule“ zu etablieren (IKTa, 223ff.).

#### Unterrichtsentwicklung

Die Erkenntnisse aus den Interviews zeigen, dass der aktuelle Einsatz von ET an der Schule weitgehend in der Übertragung klassischer Unterrichtseinheiten auf digitale Settings bestand. Künftig soll sich dies jedoch ändern:

Wenn wir digitale Settings ernst nehmen, [dann sollten] wir diese digitalen Lernsettings von vorneherein als digitale Lernsettings denken und nicht als Präsenzsettings, die anschließend ein Stück weit digitalisiert werden. (SLa, 159ff.)

Die SL beschrieb, dass neben dem bisherigen Schwerpunkt auf der (technischen) Infrastrukturebene der Blick nun auf den Unterricht und die Unterrichtsentwicklung gelegt werden solle:

Das dürfen Sie jetzt zwar nicht dem Schulträger raten, nämlich da wird weiterhin auch die Formulierung sein, dass der große Schritt weiterhin darin besteht, Hardware und Infrastruktur noch weiter auszubauen, aber wenn ich ehrlich bin, besteht die große Herausforderung darin, dass wir didaktisch und methodisch uns in digitale Lernumgebungen begeben und diese sind für uns geprägt durch Selbststeuerung und durch Selbstorganisation. Das heißt wir bewegen uns in eine andere Welt hinein. (SLa, 162ff.)

Dabei wolle man nicht auf die nächste Generation von Lehrkräften warten, denn dies wäre „doppelt naiv und blöd“ (SLa, 181). Für das Schuljahr 2021/2022 wurde dem IKT-Beauftragten zu folge ein Mediencurriculum entworfen, mit dem Ziel, die Medienkompetenz der SuS zu stärken und Unterricht entsprechend weiterzuentwickeln. Eine regelmäßige Kontrolle durch (Selbst‑)Evaluationstools wie SELFIE unterstütze die Schule, dies nachhaltig zu beobachten und gegebenenfalls anzupassen (IKT).

In Bezug auf den eigenen Unterricht wurde der durch SELFIE abgefragte Bereich der Bewertungsverfahren wiederholt aufgegriffen. Hier habe SELFIE den ein oder anderen „etwas stutzig gemacht“ (IKTb, 121f.), was mit fehlender Vorstellungskraft (IKT, LK1) und Herausforderungen beim Transfer in den digitalen Raum (LK2) in Zusammenhang gebracht wurde.

#### Personalentwicklung

Wir denken manchmal, dass wir da schon viel weiter sind, als das Kollegium eigentlich schon ist und das muss man immer wieder, glaube ich, reflektieren. Und da hilft sowas, wenn man da dann einfach so ein Blatt vornehmen kann und sagen kann, Leute so siehts aus, die Lücke muss geschlossen werden. (SEBb, 44 ff.)

Auf der Ebene der Personalentwicklung ließ sich durch SELFIE vor allem feststellen, dass Personalförderung insbesondere durch verschiedene Fortbildungsmaßnahmen zum Umgang mit und Anwendung von ET gewünscht werden (LK2, SEB, SL). Hierzu macht sich die SL Gedanken, wie sie mittels Personalfortbildungen LK bei der Implementierung von ET im Unterricht unterstützen kann:

(…) durch unterschiedliche Fortbildungsformate oder durch unterschiedliche Arrangements wie, dass Lehrer[*innen] als Gruppen zusammenarbeiten bei der Umsetzung von bestimmten digitalen Ideen. (SLa, 108ff.)

Der IKT-Beauftragte verwies im Interview auf ein „Mikrofortbildungskonzept“, mit welchem die Lehrkräfte in Form von Kurzfortbildungen bestimmte Tools oder Methodiken kennenlernten. Dabei werden praxisnahe Fortbildungen gewünscht „mit Beispielen, wie kann man sowas ganz konkret umsetzen, welche Möglichkeiten gibt es […]“ (LK2, 134f). Neben diesen Fortbildungsmaßnahmen ist im Rahmen der Personalentwicklung geplant, zunächst alle LK mit digitaler Infrastruktur (in dem Fall iPads) auszustatten, um den Umgang und die Anwendung von ET zu erlernen. Anschließend sollen die LK das neu erlangte Wissen an die SuS weitervermitteln (IKT). Auch die zuvor erwähnte Möglichkeit zur verpflichtenden Implementierung spezifischer ET (LK2) wurde als potenzielle Personalentwicklungsmaßnahme identifiziert.

#### Technologieentwicklung

Laut den Ausführungen der Befragten waren die letzten Jahre der Schulentwicklung stark durch Technologieentwicklung geprägt (IKT, M, SEVA). Somit sei die Schule auf dem aktuellen Stand (SL).

Die SL formulierte im Zusammenhang mit den Klassen- und Fachräumen zudem, dass das Ziel immer gewesen ist und auch weiterhin sei, konkrete Maßnahmen auf einen Schlag als Standard in allen Klassen- und Fachräumen umzusetzen und dabei nach Möglichkeit die Bedürfnisse des Unterrichts und der Lehrkräfte mit einzubeziehen. In der Verschränkung zwischen Technologie- und Unterrichtsentwicklung hat der IKT-Beauftragte im Interview sein Wunschdenken hinsichtlich digitaler Transformation an der Schule erklärt:

Ich würde tatsächlich jedem Schüler ein Endgerät an die Hand geben zu Schuljahresbeginn, genauso wie die Schulbücher ausgegeben werden. Ich würde ein Unterstützungssystem aufbauen für technischen Support, aber auch für den Support wie ich dieses Gerät nutze. […] auch auf Lehrerseite, dass wir da bisschen einen Support haben, der sich auch um die Erneuerung der Geräte beispielsweise kümmert, eine sehr gut ausgebaute Netzinfrastruktur, Stichwort Internetanbindung und WLAN, und eine Plattform, die das Ganze auch ermöglicht. (IKTb, 233ff.)

Politische Rahmenbedingungen hinsichtlich Gestaltungs‑, Finanzierungs- und Verwaltungsspielräumen erschweren diesen Prozess und schränken Innovationskraft ein (IKT). Eine besondere Einstellungsveränderung hinsichtlich Technologieentwicklung wurde laut SL durch die Covid-19-Pandemie ausgelöst:

Bisher gingen wir vom Bring-your-own-Device Gedanken aus, dass dieser zukünftig die Schülerausstattung prägen wird. Gerade die letzten 14 Monate haben aber gezeigt, dass es für unterrichtliche Settings sehr viel hilfreicher ist, wenn alle Schülerinnen und Schüler in einer Lerngruppe über die gleiche Hardware verfügen als mobile Endgerätausstattung. […]. Vor allem haben wir aber auch gesehen, dass es Möglichkeiten nicht nur geben muss, sondern auch geben kann, sowohl die Finanzierung als auch die Administrierung zu schultern, wenn der Schulträger bereit ist, entweder eigene Verantwortung zu übernehmen, Stichwort eigenes Personal aufzubauen, oder zum anderen wir auch neue Wege in der Finanzierung gehen, Stichwort Leasingmodelle, auch für digitale mobile Endgeräte für Schülerinnen und Schüler. (SLa, 144ff.)

Insgesamt führe die momentan in der öffentlichen Diskussion vorherrschende Berücksichtigung der Infrastrukturentwicklung zu einer „Mono-Dimensionalität“ (SLb, 107) mit welcher die Selbstevaluation „Schluss mache“ (SLb, 110), da hierdurch zwar der Bereich der Infrastruktur angemessen abgebildet sei, dieser aber „nur ein Rahmen, eine Grundlage [der digitalen Transformation] sein kann und die anderen [Bereiche] viel stärker auch den Erfolg, den Misserfolg, die Sinnhaftigkeit oder Sinnlosigkeit digitaler Bildung [deutlich machen]“ (SLb, 112ff.).

#### Kooperationsentwicklung

Die SL betont, dass das gesamte Kollegium „Willens und hochkompetent“ (SLa, 187) sei, um die digitale Transformation und künftige Implementierung von ET voranzutreiben. In diesem Zusammenhang stellt sie heraus, dass dies „in der Regel nur durch eine verstärkte Kollaboration zwischen SuS, LK und SL“ gelinge (SLa, 189f.). Hierbei wird das Potenzial von digitalen und kollaborativen Kommunikations- und Arbeitstools (wie bspw. MS-Teams) zu diesem Zwecke hervorgehoben:

Man ist weg von diesem ‚man trifft sich an einem Nachmittag‘ sondern arbeitet mehr in [Microsoft] Teams und mehr kollaboratives Arbeiten zu den Zeiten, zu denen es den Kolleg[*inn]en gut passt. Von dem her denke ich schon, dass das eine große Veränderung ist, die uns auch zukünftig begleiten wird. (IKTb, 193ff.)

Eine stärkere Kooperation fördere im Kollegium die fehlende Systematik bei der Implementierung von ET. Die Selbstevaluationsbeauftragte wünscht sich hierzu:

[…] ein Konzept, das man sagt, wir nehmen es [ET] uns so und so vor […], weil jeder beantwortet die Frage ja aus seiner eigenen Perspektive. Wenn ich aber kein Hintergrundwissen habe, beantworte ich diese natürlich anders, als wenn ich weiß, das und das und das ist der Hintergrund zu genau dieser Fragestellung. […] Wenn ich aber überhaupt keine Grundkenntnis habe, dann sage ich natürlich aufs blaue rein, klar weiß ich, wie das geht, aber ich habe nirgends eine Messlatte, dass ich das in einen Bezug setzen kann. (SEVAb, 56 ff.)

Insbesondere die festgestellte Diskrepanz der Wahrnehmung zwischen SL einerseits und andererseits LK/SuS hinsichtlich spezifischer SELFIE-Bereiche, könnte dabei mittels verstärkter Kooperation zunächst besser verstanden und aufgelöst werden (LK2).

## Diskussion

Zur übergreifenden Fragestellung hinsichtlich selbstevaluativer Schulentwicklungsprozesse in der digitalen Transformation bietet die vorliegende Case-Study Erkenntnisse auf zwei Ebenen. Zum einen wird diskutiert, inwiefern SELFIE als digitales Tool für die Selbstevaluation in Bezug auf die Implementierung von ET geeignet ist (Abschn. 5.1). Anschließend wird erörtert, inwiefern diese Selbstevaluation Auswirkungen auf die Dimensionen eines schulentwicklerischen Ansatzes zur ganzheitlichen Implementierung von ET hat (Abschn. 5.2). Abschließend erfolgen eine kritische Reflexion sowie ein Ausblick für künftige Forschung (Abschn. 5.3).

### SELFIE als Tool zur Selbstevaluation

Insgesamt erscheint die einmalige Nutzung von SELFIE aus Sicht der Interviewpartner als Tool zur Selbstevaluation für ihre Schule generell geeignet. So lässt sich mittels des automatisierten Berichts des Selbstevaluationstools ein umfangreicher Status-Quo zum Einsatz von ET in der Case-Study-Schule ableiten (vgl. FF1). In Abschn. 4.1 wurde gezeigt, dass die ausgewählte Case-Study-Schule aus Sicht verschiedener Akteursgruppen (SL, LK, Eltern) insgesamt in der – im Rahmen der Selbstevaluation analysierten Bereiche – Implementierung von ET gut aufgestellt ist. Dies wird im Abgleich mit zentralen Erkenntnissen der ICILS Studie 2013 in Bezug auf IT-Ausstattung, Akzeptanz in der Nutzung von ET sowie selbsteingeschätzte Fähigkeiten der LK bestätigt (Eickelmann et al. 2014). Neben einigen Stärken der Schule wurden auch wenige Schwächen hinsichtlich der Implementierung von ET aufgedeckt. Bei Letzteren spielte der Bereich der Bewertungsverfahren eine große Rolle. Die Erkenntnisse aus den Interviews zeigten hier eine Unsicherheit der Befragten, was das überhaupt sei und wie dies umgesetzt werden könnte. Hieraus konnte in der zweiten Interviewreihe bei allen befragten Lehrkräften eine Offenheit und Lernbereitschaft gegenüber den Schwachstellen identifiziert werden, welche nun durch gezielte Maßnahmen angegangen werden sollten. Die Verschränkung der quantitativen und qualitativen Datenanalysen ermöglichte eine kritisch-reflektierende Auseinandersetzung mit den Ergebnissen des SELFIE-Berichts, und zeigte somit auch Widersprüche auf. So gab ein Großteil der LK in der Selbstevaluation beispielsweise an, ET bei der Unterrichtsgestaltung umfangreich (76–100 % der Unterrichtszeit) anzuwenden. Den Erkenntnissen der Interviews zufolge beschränkt sich diese Anwendung jedoch größtenteils auf einen Ersatz analoger Medien mit neuen, digitalen Technologien (bspw. Smartboard oder Tablett-Einsatz anstelle des herkömmlichen Tafelanschriebs). Die positiven Ergebnisse der Selbstevaluation sollten entsprechend im Detail geprüft und im Kollegium reflektiert werden.

Die Begleitung der Case-Study-Schule mittels mehrepisodischen Interviews während des Durchführungsprozesses ermöglichte umfangreiche Erkenntnisse bezüglich Erwartungen verschiedener schulischer Akteursgruppen gegenüber Selbstevaluationstools sowie daraus angestoßener Veränderungsprozesse (vgl. FF2). Zunächst zeigte sich in der Auswertung der ersten Interviewreihe eine starke Offenheit und Akzeptanz gegenüber SELFIE als Selbstevaluationstool (Abschn. 4.2). Die Anonymität der Befragung ermöglichte dabei ein ehrliches und kritisches Feedback, was insbesondere für künftige Weiterentwicklung und Wünsche/Bedürfnisse in Richtung der SL wichtig ist.

Ein wiederkehrendes Thema in den Interviews war die durch den SELFIE-Bericht dargestellte Diskrepanz zwischen LK und SL in bestimmten Bereichen. Aufgrund fehlender Rohdaten konnte nicht überprüft werden, ob diese Subgruppenunterschiede tatsächlich signifikant und somit belastbar sind. Für eine evidenzbasierte Diskussion und Strategieentwicklung wäre dieses Wissen jedoch hochrelevant. Aus den Erkenntnissen der zweiten Interviewreihe wurde die aktive Beteiligung und Einbindung aller Akteursgruppen insgesamt als sehr positiv wahrgenommen. Durch diese Perspektiverweiterung wurden die Ergebnisse in der Wahrnehmung der Befragten positiv aufgewertet und die Selbstevaluation durch alle Akteursgruppen wohlwollend akzeptiert.

Dies bestätigt die Chancen eines ganzheitlichen und akteursübergreifenden Austauschs zentraler Akteursgruppen in Bezug auf Schulentwicklungsprozesse und die strategische Ausrichtung der Schule (Klieme 2016; KMK 2021). Dabei begünstigten und beschleunigten die Covid-19-Pandemie und der damit einhergehender Zwang, sich mit ET auseinanderzusetzen (Wohlfart et al. 2021), die Erreichung der selbst gesteckten Ziele der Schule hinsichtlich Digitalisierung.

Aus der zweiten Interviewreihe wurde jedoch deutlich, dass die Case-Study-Schule SELFIE als Selbstevaluationstool nicht langfristig in das eigene Qualitätsmanagement übernehmen wird. Dies liegt mitunter daran, dass SELFIE lediglich die digitale Leistungsfähigkeit der Schule fokussiert – jedoch keine Informationen hinsichtlich anderer, relevanter Entwicklungsbereiche abbildet. Den Erkenntnissen nach Durchführung von SELFIE folgend ist es wahrscheinlicher, dass die Schulentwicklungsgruppe spezifische Bereiche und Fragen des SELFIE-Tools in ihre eigene, bereits vorhandene und bewährte Selbstevaluation übernimmt, da diese zusätzlich zum Bereich der digitalen Transformation andere Entwicklungsbereiche abdeckt.

Die Erkenntnisse aus der Case-Study bestätigten insgesamt den aktuellen Forschungsstand, dass die alleinige (und einmalige) Durchführung der Selbstevaluation nicht ausreicht, um (nachhaltige) Veränderungsprozesse in Gang zu setzen (Eickelmann und Gerick 2017; Lorenz et al. 2017). Die Verantwortungsübernahme und Folgemaßnahmen in Reaktion auf den SELFIE-Bericht waren nach der Durchführung noch unklar, was auch die Reserviertheit gegenüber tatsächlichen Veränderungen zumindest andeutet. Hier bedarf es nun im Sinne eines kontinuierlichen und wiederholten Entwicklungsprozesses klarer nächster Schritte hinsichtlich Priorisierung und Unterstützung spezifischer Maßnahmen durch die SL zur Verarbeitung der Ergebnisse der Studie (SELFIE 2022).

### SELFIE als Teil der Schulentwicklung zur Implementierung von ET

Hinsichtlich der Dimensionen der Schulentwicklung (Abschn. 4.3) wurden Auswirkungen durch die Durchführung von SELFIE für alle fünf vorgestellten Dimensionen der Schulentwicklung identifiziert (vgl. FF3). Demnach spielt im Rahmen der Organisationsentwicklung insbesondere die künftige Implementierung und Nutzung digitaler Tools für die Verwaltung und Organisation der Schule eine wichtige Rolle. Die SL plädierte für ein hohes Maß an Entscheidungshoheit über Einsatzbereiche und Nutzung durch die Einzelschule. Bürokratisch aufwendige Verfahren wie die Verwaltungsvereinbarung DigitalPakt Schule 2019–2024 (Wohlfart und Wagner im Druck) erscheinen demnach eher ungeeignet für die individuellen Bedürfnisse innerhalb der Organisationsentwicklung der Case-Study-Schule.

Die Unterrichtsentwicklung nimmt an der analysierten Schule den Ergebnissen zufolge im Rahmen der digitalen Transformation die bedeutsamste Rolle ein. Diese lässt sich jedoch nicht von der fortgeschrittenen Entwicklung anderer Bereiche isoliert betrachten: die Schule ist beispielsweise hinsichtlich digitaler Infrastruktur hervorragend ausgestattet, sodass der Blick künftig auf die didaktisch sinnvolle Implementierung von ET im Unterricht gerichtet werden kann. Auch die Personalentwicklungsdimension ist hiermit eng verknüpft: die Befragten der Schule wünschten sich konkrete und praxisnahe Fortbildungsmaßnahmen zum Umgang mit und zur Anwendung von ET im Unterricht. Hierbei kam in den Interviews auch zur Sprache, dass LK nicht zu ihrem eigenen, sondern zum Nutzen der SuS kompetent im Umgang mit digitalen Medien sein sollen, um diese auf die digital geprägte Welt vorzubereiten (BMBF 2016; EU 2020, 2021; KMK 2016). Es erscheint dabei sinnvoll, neben einer allgemeinen Digitalkompetenz der LK, fachspezifische sowie berufsbezogene Digitalkompetenz im Rahmen von Personalentwicklungsmaßnahmen zu vermitteln (Pettersson 2018). Hierzu wurde von einem Befragten beispielsweise die Verpflichtung zur Implementierung (spezifischer) ET im Rahmen des Unterrichts als potenzielle Personalentwicklungsmaßnahme vorgeschlagen. Im Sinne eines Empowerment-Ansatz in der Schulentwicklung (Copland 2003; Schildkamp et al. 2016) sollte dieser Schritt nicht von der SL, sondern bestenfalls im Rahmen einer offenen und akteursübergreifenden Diskussionsrunde partizipativ entschieden werden. Die SL scheint sich dessen auch bewusst und plädiert für eine verstärkte Kollaboration zwischen den Akteursgruppen, wie auch durch Eickelmann und Gerick (2017) in Bezug auf digitale Transformationsprozesse explizit empfohlen. Die SL der Case-Study-Schule betonte abschließend in Bezug auf Technologieentwicklung die Vielschichtigkeit der Implementierung von ET und bemängelte die „Mono-Dimensionalität“ in der öffentlichen Diskussion hinsichtlich Infrastrukturentwicklung als Patentrezept für eine erfolgreiche digitale Transformation.

Die Erkenntnisse aus den Interviews bestätigen die Wechselwirkungen zwischen den einzelnen Entwicklungsdimensionen. Für eine ganzheitliche Implementierung von ET in der Schule (BMBF 2016; KMK 2016, 2021), bedarf es somit wie von Eickelmann und Gerick (2017) gefordert, einer Erweiterung des bisherigen Verständnisses der Schulentwicklung nach Rolff (2010, 2016), um die Entwicklungsdimensionen der Technologie- und Kooperationsentwicklung. Die Erkenntnisse der Studie zeigen enge Verknüpfungen dieser Dimensionen und betonen dabei die tragende Rolle der SL in diesem Prozess. Aktuelle Studien im Bereich der Schulentwicklung greifen diesen Gedanken der „Gatekeeper-Funktion“ der SL bereits auf (Dexter 2018; Heinen und Kerres 2017; Tulowitzki und Gerick 2020). Vonnöten sind den Erkenntnissen hieraus folgend nicht nur (finanzielle und zeitliche) Förderungen der LK im Rahmen von spezifischen Fortbildungsmaßnahmen; vielmehr sollte die SL alle Akteursgruppen partizipativ einbinden, ihre Rolle als Vorbild hinsichtlich der Implementierung von ET im Unterricht sowie in der Verwaltung und Kommunikation einnehmen und Eigeninitiative von LK sowie SuS explizit fördern bzw. unterstützen.

### Limitationen und Ausblick

Abschließend wird kritisch auf den Beitrag der vorliegenden Studie sowie das Potenzial von SELFIE für die Schulentwicklungsforschung geschaut. Aus Praxisperspektive hat die Durchführung und Begleitung von SELFIE klare Vorteile und Chancen für die Case-Study-Schule (siehe Abschn. 5.1). Daraus entstehen praktische Implikationen für die Einzelschule, welche eine ganzheitliche, digitale Transformation unterstützen. Aus Forschungsperspektive sind jedoch auch Limitationen gegeben.

Zunächst muss berücksichtigt werden, dass in der Studie nur eine Schule betrachtet wurde. Es ist jedoch nicht das Ziel einer Case-Study, allgemeingültige Aussagen zu treffen. Vielmehr wird anhand eines ausgewählten Falls ein zeitgenössisches Phänomen eingehend in seinem realen Kontext aus verschiedenen Perspektiven untersucht (Yin 2018). Vor dem Hintergrund fehlender Forschungserkenntnisse zur digitalen Transformation von Schulen, der Dynamik dieses Entwicklungsprozesses sowie aktuellen Forderungen der KMK (2021), diese auf Einzelschulebene in den Blick zu nehmen, leistet der vorliegende Beitrag somit einen ersten Schritt in diese Richtung. In weiteren Schritten sollte eine kritische Reflexion der Abhängigkeiten und Zusammenhänge des Bildungssystems und einer möglichen (Einzel‑)Schulentwicklung mit den politischen und ökonomischen Systemen folgen.

Im Transfer der Erkenntnisse auf andere Schulen sollte neben dem aufgezeigten Status-Quo der untersuchten Schule zusätzlich berücksichtigt werden, dass es sich im Falle dieser um eine Berufsschule handelt, welche in der Regel einige Unterschiede gegenüber allgemeinbildenden Schulen der Primar- und Sekundarstufe hinsichtlich Größe und Abteilungen sowie verschiedener Bildungsgänge aufweist. Ein Transfer der Erkenntnisse steht zudem im Kontrast zu den eigentlichen Zielen von SELFIE, welches als praktisches Tool für Einzelschulen konzipiert wurde und Vergleiche über Schulen hinweg ausschließt (SELFIE 2022).

Abschließend stellt sich die Frage, ob und wie SELFIE somit als Selbstevaluationstool einen Beitrag zur Schulentwicklungsforschung leisten kann. Das Tool wurde konzipiert und validiert, um Schulen mit unterschiedlichen Niveau- und Ausprägungsstufen hinsichtlich ihrer digitalen Leistungsfähigkeit gerecht zu werden. Damit kommt SELFIE zumindest in Teilen politischen und wirtschaftliche Forderungen einer digitalen Transformation des Bildungssystems nach und empowered die Einzelschulen in ihrer individuellen Schulentwicklung. Während psychometrische Analysen des Tools mittels transnationaler Daten dessen Eignung zur Selbstevaluation hinsichtlich Reliabilität und interner Konsistenz bestätigen (Costa et al. 2021), wäre der Zugang zu den anonymisierten Rohdaten im Sinne der Open Science Richtlinien (EU 2019) sowohl für die Einzelschulen sowie die Wissenschaftscommunity wünschenswert, um eine Datenauswertung nach wissenschaftlichen Standards (z. B. Signifikanzüberprüfung vorhandener Subgruppenunterschiede) leisten sowie evidenzbasierte Strategien einer nachhaltigen Schulentwicklung ableiten zu können.

## Anhang

**Table 3:**

Hauptkategorie	Definition Hauptkategorie	Unterkategorien	Definition Unterkategorien	Ankerbeispiel
Schulentwicklung und Schulentwicklungsdimensionen	Aussagen, welche einen Bezug zur Schulentwicklung allgemein oder einer Schulentwicklungsdimension speziell nehmen	Schulentwicklung allgemein	Aussagen, welche sich allgemein auf Schulentwicklung beziehen	„momentan kämpfen wir immer mehr damit, […] bei den Entscheidungsträgern die letztendlich über die Verwendung der Mittel entscheiden unsere Sichtweise, was bedeutsam ist für die weitere [Schul-]Entwicklung […] ankommen zu lassen.“ (SLa)
		Unterrichtsentwicklung	Aussagen, welche sich auf die Weiterentwicklung des Unterrichts beziehen	„Wir sind momentan in dem Stadium, […], dass wir primär versuchen, einen klassischen, gelernten Präsenzunterricht soweit wie möglich auch im Setting in die digitale Welt zu verlagern. […] Das ist aber noch nicht meine Vorstellung oder mein Verständnis von digitalbasierten Lehr und Lernsetting. Hier müssen wir uns noch deutlich weiterentwickeln auch in Bezug auf die Didaktik.“ (SLb)
		Organisationsentwicklung	Aussagen, welche sich auf strukturelle und organisationale Weiterentwicklung der Schule beziehen	„[…] Schulentwicklung ist oft so ein bisschen problemorientiert und dann wird da drüber diskutiert und es wird die Ursache gesucht, anstatt einfach mal Sachen auszuprobieren und zu gucken, ob das jetzt funktioniert. Und bei einem großen Kollegium wie wir hier jetzt sind, wir sind ja 140 Leute, da kann man nicht an einem Thema oder einem Curriculum arbeiten, das ist zu viel. […] eigentlich wärs gut wenn man kleine Gruppen hat die dann einfach mal was bestimmen und das wird dann getestet und durchgezogen und dann kann man in einem Jahr immer noch gucken, hats überhaupt nicht funktioniert, oder doch oder nur ein bisschen, bauen wir es weiter aus, lohnt sich das oder nicht.“ (LK1)
		Personalentwicklung	Aussagen, welche sich auf die, das gesamte Personal der Schule betreffende, Weiterentwicklung beziehen	„in der Theorie funktioniert es sehr gut […], denn wir haben alle Möglichkeiten, aber an der Umsetzung hapert es tatsächlich noch und ich glaube da bräuchte man halt irgendwie noch konkretere Fortbildungsmaßnahmen“ (LK2)
		Technologieentwicklung	Aussagen, welche sich auf die Weiterentwicklung von Schule und/oder Unterricht hinsichtlich Technologie beziehen	„… dass man sich jetzt überlegt das man bis vor zwei Jahren keine ordentliche digitale Infrastruktur hatte an der Schule, wenn man sich jetzt überlegt zwei Jahre später was ist passiert, dann ist das für mich ein großer Schritt der passiert ist und das läuft auch gut“ (LK2)
		Kooperationsentwicklung	Aussagen, welche sich auf die Weiterentwicklung von Kooperationen zwischen und innerhalb schulischen Akteursgruppen beziehen	„Ich denke eine Systematik müsste her. Bisher wurschtelt jeder für sich vor sich hin, aber es gibt in meinen Augen, zumindest hab ich’s noch nicht gesehen, keine Systematik“ (SEBb)
		5‑Jahres-Plan	Aussagen, welche sich auf den schulinternen 5‑Jahresplan beziehen	„Gerade da ist unser großes fünf Jahres Ziel Digitalisierung bzw. die Möglichkeiten, die die Digitalisierungswelle bietet, gerade fürs Lehren und Lernen zu nutzen.“ (IKTa)
Digitalisierung	Aussagen die sich auf Aspekte von Digitalisierung und digitaler Transformation beziehen	Möglichkeiten	Aussagen, die sich auf Möglichkeiten durch Digitalisierung beziehen	„Die Stärken wären, dass wir gerade durch die Digitalisierung auch Fernlernunterricht anbieten können bzw. auch bei Schülern in besonderen Situationen diese zuschalten können. Muss ja jetzt nicht immer coronabedingt sein, sondern kann ja auch sein wir haben ja ab und zu auch Schüler, die krankheitsbedingt länger weg sind. Diese hätten dann eine Möglichkeit am Unterricht teilzunehmen.“ (SEVAa)
		Risiken	Aussagen, welche Gefahren oder Risiken sich durch Digitalisierung bieten	„Das wäre glaube ich der größte Fehler den man begehen kann zu formulieren es geht primär darum digitale Medien einzusetzen, umzusetzen und einzubringen. Digitalisierung und digitale Medien sind unverändert Medien, das heißt, sie sind nicht Ausgangspunkt einer Entscheidung sondern sie sind Medien die der Erreichung eines Ziels dienen müssen, die aber einen unglaublichen Medienraum eröffnen.“ (SLa)
		Hürden/Hindernisse	Aussagen, welche Hürden/Hindernisse im Digitalisierungsprozess auftreten können	„es fehlen Rechner, Drucker und Zugänge das man halt auch einfach was arbeiten kann, wenn man die Arbeitsgeräte nicht dabei hat. Oder, der Trend geht ja hin zu BYOD [Bring your own Device], dass sie dann aber die Möglichkeit haben wenigstens über WLAN-Nutzung oder Internetnutzung irgendwas tun [können].“ (SEVAa)
		Gelingensbedingungen	Aussagen, welche beschreiben, was nötig ist damit digitale Lehre und digitales Lernen zukunftsfähig wird	„… Umstand der momentan in der öffentlichen Diskussion eine Mono-Dimensionalität mit sich bringt, nämlich die Reduktion auf die Infrastruktur. Also ‚plump‘ formuliert, wenn alle mal ein iPad in der Hand haben und wenn vorne ein Smartboard installiert ist, dann ist digitale Bildung hergestellt und quasi dieses Vorhaben abgeschlossen. Mit dieser sehr naiven Vorstellung macht spätestens SELFIE Schluss, weil natürlich der Bereich der Infrastruktur, er wird abgebildet, aber es wird mehr als deutlich, dass das nur ein Rahmen, eine Grundlage sein kann und die anderen viel stärker auch den Erfolg, den Misserfolg, die Sinnhaftigkeit oder Sinnlosigkeit digitaler Bildung prägenden Fragen deutlich werden.“ (SLb)
		Digitale Entwicklung	Aussagen, welche sich auf den Wandel mit und durch digitale Technologien beziehen	„Ich denke Schulentwicklung wird ohne Digitalisierung in den nächsten Jahren nicht passieren.“ (LK2)
		Implementierung von ET	Aussagen über den Zugang zu oder die Implementierung von ET in Schule/Unterricht	„Ich würde tatsächlich jedem Schüler ein Endgerät an die Hand geben zu Schuljahresbeginn genau so wie die Schulbücher ausgegeben werden. Ich würde ein Unterstützungssystem aufbauen für technischen Support aber auch für den Support wie ich dieses Gerät nutze. Was es mir bringen kann, wie ich da mein Lernen optimieren kann und wie ich da mit andern zusammenarbeite. Das wäre auf jeden Fall nicht nur auf Schülerseite mein Wunsch sondern natürlich auch auf Lehrerseite, dass wir da bisschen einen Support haben, der sich auch um die Erneuerung der Geräte beispielsweise kümmert, eine sehr gut ausgebaute Netzinfrastruktur, Stichwort Internetanbindung und WLAN und eine Plattform, die das ganze auch ermöglicht. Also das Arbeiten und die stabil und performant läuft.“ (IKTa)
Selbstevaluation	Aussagen, die sich auf Selbstevaluation und Selbstevaluationsprozesse beziehen	Erfahrung mit Selbstevaluationstools	Aussagen, welche sich auf bisherige Erfahrungen der Interviewpartner*innen bezüglich Selbstevaluationsinstrumente beziehen	„Ja also wir betreiben das ja schon seit Jahren und wir haben schon mehrere Umfragen im Kollegium aber auch in der Schülerschaft“ (IKTa)
		Probleme	Aussagen, welche sich auf Probleme bzw. Schwierigkeiten mit Selbstevaluation beziehen	„Die Akzeptanz ist im Kollegium allerdings relativ gering.“ (SEBa)
		Wirkung	Aussagen, welche sich auf die Auswirkung von Selbstevaluation beziehen	„das sollte vielleicht der Zweck dahinter sein […], das eben eine Selbstevaluation etwas positives ist […], was mir etwas gibt und was mich nicht irgendwie abstrafen will sondern wirklich etwas gibt.“ (M)
SELFIE	Aussagen, welche sich spezifisch auf das Selbstevaluationstool SELFIE beziehen	Möglichkeiten	Aussagen, welche Chancen/Möglichkeiten SELFIE bietet	„Ich mache einmal den IST Zustand, dann lese ich darauf ab wo ich Handlungsbedarf habe, handle dann auch in einem bestimmten Umfang und überprüfe dann, ob mein Handeln auch das gezeigt hat bzw. das entsprechend verändert, was ich damit verändern wollte. Also wenn ich sowas einsetze, muss ich es wirklich über einen längeren Zeitraum machen damit ich auch die Entwicklung betrachten kann.“ (SEVAa)
		Stärken	Aussagen bezüglich identifizierten Stärken von SELFIE	„Also sinnvoll fand ich auf jeden Fall das es aufgeteilt wurde in Schulleitung, Lehrer und Schüler.“ (LK1)
		Schwächen	Aussagen bezüglich identifizierten Schwächen von SELFIE	„Wie gesagt, es ist ein gutes Tool. Was ich denke ist, dass es eben nicht so flexibel anpassbar ist wie jetzt unsere selbstgestrickte SEVA. Hier können wir halt spezifisch unsere Jahresziele integrieren und abfragen, wir haben dann auch immer die Atmosphärischen Sachen drinnen.“ (SEBb)
		Veränderungsprozesse	Aussagen, bezüglich Veränderungsprozessen an der Schule im Zusammenhang mit SELFIE	„SELFIE [ist] ein Medium […] und kein Selbstzweck. Das heißt auch hier werden wir schauen müssen, worin bestehen unsere Ziele und wobei kann uns Selfie, wie klassischer Weise auch ein Selbstevaluationsinstrument helfen? Durch das liefern von Daten um einen Ist-Zustand darzustellen und darauf aufbauend zu wissen, wo stehen wir gut, wo wollen wir hin und wie können wir das erreichen“ (SLa,)
		Gelingensbedingungen	Aussagen, welche im Zusammenhang mit dem Gelingen von SELFIE an der Schule verknüpft sind	„Vielleicht könnte man das Kollegium doch etwas mehr mit einbeziehen, also das ganze etwas breiter gefächert. Und da vielleicht auch mal drauf bauen, dass gerade bei solchen Sachen eine höhere Teilnehmerquote zustande kommt.“ (LK2)
		Hürden/Hindernisse	Aussagen bzgl. Problemen, welche vor/während/nach der Durchführung von SELFIE auftreten können	„Also wünschen würde ich mir, dass es sehr niederschwellig ist für die Benutzer und auch für die Auswertung. Niederschwellig, Komfortabel. Was schön ist, ist wenn so ein Tool in einem hohen Maße individualisierbar ist. Momentan habe ich da den Eindruck, das ist bei SELFIE nicht so“ (SEBa)
		Bekanntheit	Aussagen bzgl. der Bekanntheit von SELFIE	„Sie meinen im Vorfeld bevor sie uns das angetragen haben, nein.“ (SEBa)
		Erwartungen	Aussagen bzgl. (negativer oder positiver) Erwartungen zu SELFIE	„Ich gehe da ziemlich ergebnisoffen dran. Also ich bin gespannt wie es im Vergleich zu unserer Befragung gerade im Hinblick auf Digitalisierung vielleicht neue Ideen und neue Ansatzpunkte für die weitere Verbesserung bringt.“ (IKTa)
		Verbesserungsbedarf	Aussagen bzgl. geäußerten Verbesserungsvorschlägen zu SELFIE	„Was ich da vielleicht vermisse an SELFIE, dass man tatsächlich da hinter diesen Kompetenzen und Abfragen, die man da macht vielleicht auch Ideen und Vorschläge unterbreitet was man denn da nutzen kann. Also wenn da steht Nutzung von Daten zur Lernverbesserung (…) kann man da vieles drunter verstehen, aber wenn man da jetzt irgendwie einen Vorschlag hat oder ein Beispiel oder eine Best Practice hat dann […] wäre das hilfreich für die Schulen.“ (IKTb)
		Empfindung Fragebogen	Aussagen zur emotionalen Empfindung während des Ausfüllens des SELFIE-Fragebogens	„Ja also an manchen Stellen wusste ich nicht oder habe ich mich gefragt, wie zielführend das ist und an anderen Stellen war es mir ganz klar.“ (LK2)
Ergebnisse SELFIE Bericht	Aussagen, welche sich auf die Ergebnisse des SELFIE Berichts beziehen	Zukünftiger Nutzen	Aussagen, welche sich mit der weiteren Nutzung der Ergebnisse beschäftigen	„die müssen [die Ergebnisse] jetzt in der Schulentwicklungsgruppe […] diskutieren, die müssen sich auch ganz konkret die einzelnen Fragen und Antworten […] auch im ausführlichen Bericht […] um zu sehen wo sind die Schwierigkeiten und sich dann überlegen natürlich, wo man weiterarbeiten kann und was auch Sinn für die Schule macht daran weiter zu arbeiten, was wird sich vielleicht jetzt auch in den nächsten Jahren lohnen da mehr Aufwand reinzustecken und so weiter.“ (LK2)
		Veränderung Perspektive	Aussagen, welche sich auf die Veränderung der persönlichen Perspektive zur digitalen Leistungsfähigkeit der Schule durch SELFIE beziehen	„Wir denken manchmal, dass wir da schon viel weiter sind, als das Kollegium eigentlich schon ist und das muss man immer wieder glaube ich reflektieren. Und da hilft sowas, wenn man da dann einfach so ein Blatt vornehmen kann und sagen kann, Leute so siehts aus, die Lücke muss geschlossen werden“ (SEBb)
		Entwicklungspotenzial	Aussagen, welche ein persönliches Entwicklungspotenzial betreffen	„Also ich habe meinen eigenen Unterricht natürlich reflektiert. Habe auch wieder erkannt das ich einige Inhalte gar nicht oder unzureichend da berücksichtige. Ich habe aber auch Ideen bekommen […] das man seinen eigenen Lernzuwachs oder das man zum Beispiel messen kann wie oft man mündlich was gesagt hat“ (LK1)
		Negativ	Aussagen, welche negative Eigenschaften des SELFIE-Berichts behandeln	„Also immer wieder hatte ich quasi dieses Bauchgefühl, dass ganze lässt ein Stückweit an Substanz vermissen. In der Hinsicht, wenn ich mich anschließend bei den Ergebnissen relativ elegant herauswinden möchte, wird es mir leicht fallen mich herauszuwinden bzw. mir das Ergebnis so hinzukonstruieren, wie ich es gerne hätte um es zu benötigen. Da fehlt mir manchmal die Verbindlichkeit und Klarheit gleichermaßen aber wissend wie schnell die Grenzen eines solchen Tools erreicht sind“ (SLb)
		Positiv	Aussagen, welche positive Eigenschaften des SELFIE-Berichts behandeln	„Also die Darstellung ist auf jeden Fall super anschaulich. Das finde ich wichtig. Also sinnvoll fand ich auf jeden Fall das es aufgeteilt wurde in Schulleitung, Lehrer und Schüler. Ich glaube wenn das nicht gewesen wäre, wäre es absolut nicht aussagekräftig gewesen.“ (LK1)
		Diskrepanz	Aussagen, welche sich auf die Diskrepanzen der Ergebnisse zwischen den verschiedenen Befragungsgruppen beziehen	„interessant ist, […] dass die Schulleitung die Situation besser einschätzt als Lehrer und Schüler. […] da muss dann irgendwo eine Diskrepanz sein bei der Wahrnehmung und das finde ich schade, weil ich habe schon das Gefühl im Lehrerkollegium es war schon erschlagend die letzten beiden Jahre, man stand vor einer großen Hürde, hat versucht sein Bestes zu geben und das irgendwie zu machen und ich glaube aber man ist nicht zu 100 % zufrieden ganz klar und das finde ich witzig, dass das dann auf Schulleitungsebene alles als super positiv empfunden wird […]“ (LK2)

## Footnotes

1 Der Bereich Schulleitung bezieht sich beispielsweise „auf die Rolle der Schulleitung bei der schulweiten Integration von digitalen Technologien und ihrer wirksamen Nutzung für die Kernfunktionen der Schule: Lehren und Lernen“ (SELFIE 2021, S. 36) und umfasst die gleichen sechs Items für SL und LK zur Einschätzung der Zufriedenheit hinsichtlich der (1) digitalen Strategie der Schule, (2) Strategieentwicklung mit den LK, (3) Neue Unterrichtsformen, (4) Zeit zur Erforschung des digitalen Lehrens, (5) Urheberrechts- und Lizenzvorschriften sowie (6) die Einbeziehung von Unternehmen in die Strategie. Eine Übersicht der einzelnen Fragen sowie Ausformulierung der Items ist auf der offiziellen SELFIE-Website ersichtlich: https://bit.ly/3I6HwYp.
